# Joint Modelling of Latent Cognitive Mechanisms Shared Across Decision-Making Domains

**DOI:** 10.1007/s42113-023-00192-3

**Published:** 2024-01-11

**Authors:** Niek Stevenson, Reilly J. Innes, Russell J. Boag, Steven Miletić, Scott J. S. Isherwood, Anne C. Trutti, Andrew Heathcote, Birte U. Forstmann

**Affiliations:** Department of Psychology, University of Amsterdam, Amsterdam, Netherlands

**Keywords:** Decision-making, Cognitive neuroscience, Joint modelling, Bayesian factor analysis

## Abstract

Decision-making behavior is often understood using the framework of evidence accumulation models (EAMs). Nowadays, EAMs are applied to various domains of decision-making with the underlying assumption that the latent cognitive constructs proposed by EAMs are consistent across these domains. In this study, we investigate both the extent to which the parameters of EAMs are related between four different decision-making domains and across different time points. To that end, we make use of the novel joint modelling approach, that explicitly includes relationships between parameters, such as covariances or underlying factors, in one combined joint model. Consequently, this joint model also accounts for measurement error and uncertainty within the estimation of these relations. We found that EAM parameters were consistent between time points on three of the four decision-making tasks. For our between-task analysis, we constructed a joint model with a factor analysis on the parameters of the different tasks. Our two-factor joint model indicated that information processing ability was related between the different decision-making domains. However, other cognitive constructs such as the degree of response caution and urgency were only comparable on some domains.

## Introduction

Decision-making is a critical part of everyday life which underpins many types of actions. Even though there is a large range of decisions that individuals regularly engage in, researchers posit that various decision-making processes can be described within the framework of evidence accumulation models (EAMs; Donkin & Brown, 2018; Forstmann et al. , 2016).

EAMs posit that decision-makers gather information for each choice alternative until sufficient evidence for one alternative has been accumulated to commit to a decision (Donkin & Brown, 2018; Ratcliff & Smith, 2004). Although many implementations of EAMs exist, most propose that decision-making is governed by a combination of at least three latent cognitive processes. First, the drift rate drives the speed of the evidence accumulation process. Second, the threshold captures the amount of evidence needed to commit to a decision. Third, the non-decision time comprises both the time necessary for the sensory processing of the stimulus and the time taken to execute the motor response. EAMs describe response times and choices simultaneously, providing a model that accounts for both modalities of observed decision-making behavior. Given the success of EAMs in describing decision-making, and their sensitivity to manipulations of the decision-process, their use is widespread (Ratcliff et al., 2016). EAMs are applied to study decision-making across various domains, such as conflict processing, perceptual decision-making, value-based decision-making, learning, working memory, and the speed-accuracy trade-off (e.g., Miletić et al. , 2021; Rae et al. , 2014; McDougle & Collins, 2020; Hübner & Schlösser, 2010; Polanía et al. , 2014; Boag et al. , 2022).

To accommodate the differences between these tasks within the framework of evidence accumulation, EAMs are often adjusted for task-specific processes (e.g., Boag et al. , 2019; McDougle & Collins, 2020; van Ravenzwaaij et al. , 2019). Furthermore, cognitive psychologists assume that the type of evidence accumulated differs between types of decisions. For example, the strength of accumulation for decisions involving working memory is assumed to be based on the strength of working memory representations (Boag et al., 2021), whereas the accumulation process for value-based decision-making may depend on subjective preference for alternate values (Ratcliff et al., 2016). Nevertheless, the underlying architecture of accumulating evidence for various choice options until a threshold is met remains the same. Thus, explicit links between these designs exist in the framework of EAMs. If, for example, a subject is generally fast to respond due to a lack of caution, this is assumed to hold for different tasks (Hedge et al., 2019). Furthermore, even though the type of accumulated evidence differs between tasks, the accumulation process is still assumed to be based on information processing ability, a trait that should show similarities between tasks (Weigard & Sripada, 2021).

Previous studies have tested the assumption that the cognitive processes assumed by EAMs are related between different decision-making tasks (Lerche & Voss, 2017; Lerche et al., 2020; Weigard et al., 2021; Yap et al., 2012; Schulz-Zhecheva et al., 2016; Ratcliff & Childers, 2015; Schmiedek et al., 2007). These studies mostly found that an individuals’ ability to discriminate information efficiently, as measured by the most prominent EAM, the DDM, was related between different decision-making tasks. Still, these studies relied on tasks that engaged similar decision-making processes, for example, cognitive control.

Here, we chose to study tasks within a wider range of domains of decision-making tasks, keeping in mind that these domains may engage different regions across the brain. We were specifically interested in tasks that are known to engage both cortical and subcortical regions, which include the domains of working memory (Rac-Lubashevsky & Frank, 2021), value-based decision-making and reinforcement learning (O’Doherty, 2004; Gläscher et al., 2010), balancing speed and accuracy (Bogacz et al., 2010; Forstmann et al., 2008), and cognitive control/conflict tasks (de Hollander et al., 2017; Miletić et al., 2020; Aron & Poldrack, 2006; Isherwood et al., 2022). Studying these broader domains also allows us to assess whether the parameters in the EAM framework are stable across domains for which the neural underpinnings might partly vary.

To that end, we relied on four different decision-making tasks that engage different domains of decision-making: (1) a reversal learning task (RL-Rev; Costa et al. , 2015; Behrens et al. , 2007; Miletić et al. , 2021), (2) the multi-source interference task (MSIT; Bush et al. , 2003), (3) the reference-back task (RB; Rac-Lubashevsky & Kessler, 2016a; Rac-Lubashevsky & Kessler , 2016b), and (4) a reinforcement learning speed-accuracy trade-off task (RL-SAT; Sewell et al. , 2019; Miletić et al. , 2021).

**Fig. 1:**
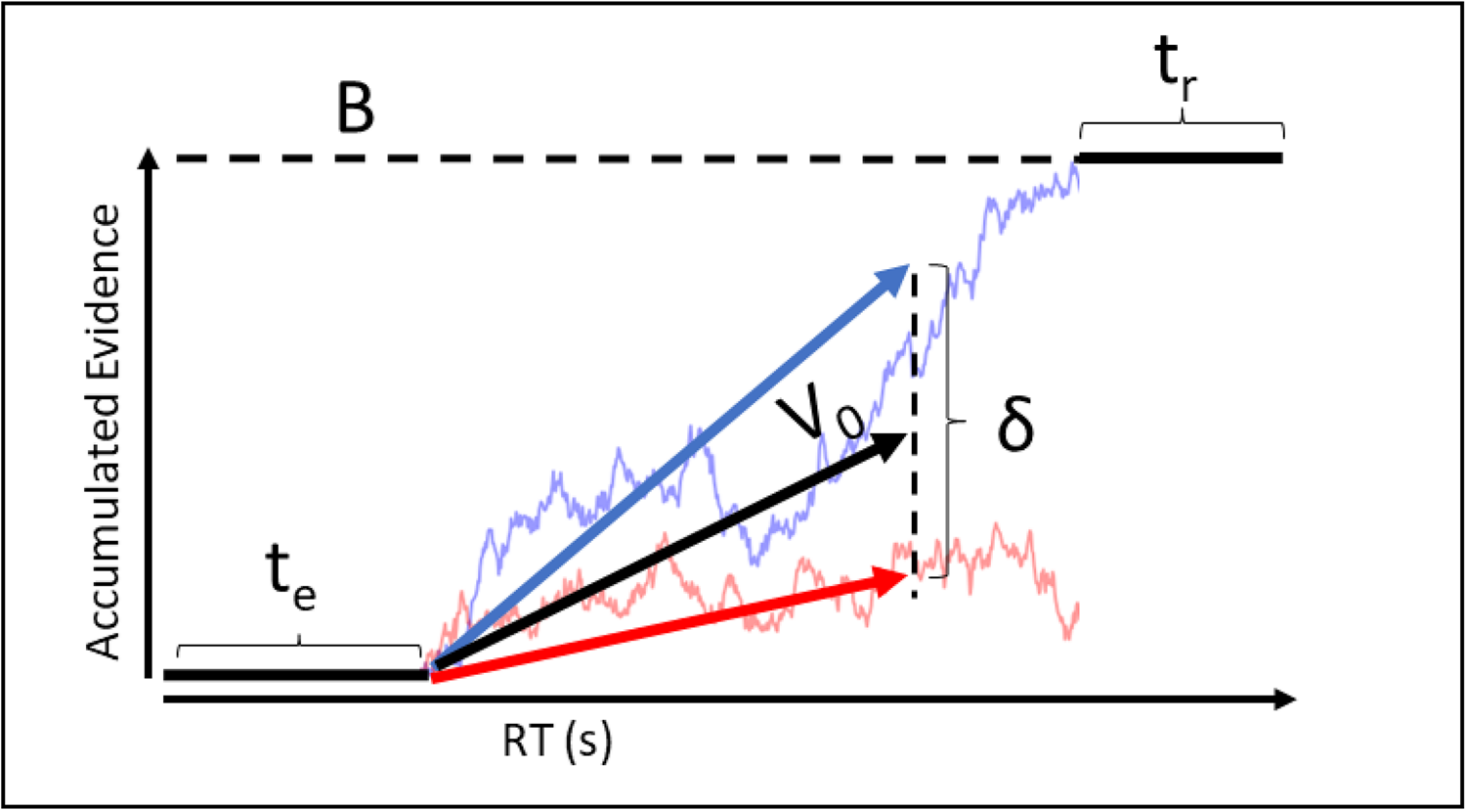
A graph of the racing diffusion model (RDM). Competing accumulators race towards threshold (*B*). The straight blue and red lines indicate the mean drift rate of two accumulators. The noisy lines indicate potential accumulation paths subject to within-trial noise in evidence accumulation. The response is determined by the first accumulator to reach the threshold. The response time is determined by the time taken to reach the threshold, plus the non-decision time ($$t_0)$$. $$t_0$$ constitutes both encoding time ($$t_e$$) and response execution time ($$t_r$$). However, these cannot be disentangled based on behavioral data alone and are therefore estimated as one parameter. In the current paper, we reparameterize the drift rates of the two choices as a mean drift rate ($$V_0)$$ and a difference in drift rate ($$\delta $$). This mean can also be interpreted as an evidence-independent urgency signal, and the difference can be interpreted as the information processing ability of the decision-maker

The four tasks involve different domains of decision-making; however, all four tasks were forced choice speed decision-making tasks that fit within the EAM framework (Donkin & Brown, 2018). All task-specific EAMs contained response threshold parameters and non-decision time parameters. Furthermore, for each task, we reparameterized the drift rate as a combination of an evidence-dependent part of the accumulation process, which is characterized by information processing ability, and an evidence-independent part of the accumulation process, which can be characterized as urgency (see Fig. 1; Miletić & van Maanen, 2019). Both urgency and response caution allow decision-makers to strategically adjust their response speed; however, they have unique contributions to the decision-process. Increased urgency only leads to an increased probability of making a response with passing time, whereas higher response caution gives more time to choose the correct stimulus (Trueblood et al., 2021; Miletić & van Maanen, 2019).

Besides engaging a broader scope of decision-making domains, the current study also uses a new methodology to test the relationships between these domains. Most previous studies that investigated the extent to which EAM constructs were related between different tasks, correlated the parameters of the EAMs to each other in a second, independent, step of analysis. However, recent work has highlighted that in order to best account for any relations that may exist between cognitive measures of different tasks, these relations should be explicitly accounted for in the model itself. Consequently, the model also accounts for measurement error and uncertainty within the estimation of these relations (Matzke et al., 2017; Turner et al., 2013; Wall et al., 2021).

To elaborate, cognitive models are often estimated in a Bayesian hierarchical framework, in which the cognitive model parameter estimates of individual subjects are related through an overarching group distribution of each parameter. Besides having desirable effects on the estimation of the parameters of the model (Rouder & Lu, 2005; Scheibehenne & Pachur, 2015), such a group-level distribution is also beneficial since it facilitates inference at the level which is usually the target for analysis in psychological science—the population. In most studies that aim to investigate links between different decision-making tasks, different EAMs are estimated independently, in that no relationships between the different group-level parameters of these EAMs are estimated. As mentioned above, the correlations between parameters are then calculated afterwards.

However, recent advances have used a multivariate normal distribution to describe the group level, which allows an explicit account of the covariances between the group-level parameters (Turner et al., 2013; Gunawan et al., 2020). Including a covariance structure in the group level allows us to estimate the likelihood of multiple models (components) simultaneously, by simply extending the vector of parameters to be estimated, while explicitly allowing the parameters (within and between components) to inform one another through the covariance structure. We refer to this approach that explicitly accounts for relationships between parameters as the joint modelling covariance approach (Turner et al., 2017).

Joint modelling of cognitive model parameters of different tasks has two advantages compared to standard correlational approaches in which the correlations are calculated after the models were fit individually. First, estimation precision is improved, and less attenuation of the true correlations is achieved by explicitly allowing relationships between parameters within the model, since measurement noise and parameter uncertainty are accounted for (Matzke et al., 2017; Rouder et al., 2019; Wall et al., 2021). Second, since the covariances are also estimated in a Bayesian approach, it is possible to construct credible intervals of correlation estimates, providing us with an inherent estimate of the uncertainty of our inferences.

Building on the covariance approach, recent work suggested to replace the multivariate normal distribution at the group level, with a factor analysis decomposition (Turner et al., 2017; Kang et al., 2021; Innes et al., 2022). This approach reduces the number of estimated parameters in the joint model, which can improve estimation with multiple components included in the joint model, yet still captures the relationships that exist between these parameters. Furthermore, with the hierarchical factor model, we estimate latent factors that span the decision-making process across different decision-making domains and can aid interpretation. Rather than inspecting separate correlation estimates, these latent factors can be interpreted across tasks. One factor with high threshold parameter loadings across tasks would for example indicate that response caution is related across tasks.

In this study, we used the hierarchical factor modelling approach to investigate the relationships between the aforementioned four decision-making tasks. The participants completed each task twice in different sessions. Therefore, we could first test to what extent decision-making was related between different time points. More importantly, since the four tasks were completed by the same set of participants, we could test whether the latent cognitive processes underlying decision-making, as proposed by EAMs, were related between different decision-making domains.

## Materials and Methods

### Subjects and Procedure

A total of 150 students from the University of Leiden, the Netherlands, participated in an experiment consisting of five tasks that were each to be completed twice online from their own computers. The study was approved by the local ethics committee. The order in which the tasks and sessions were completed was counterbalanced between participants. Each session was separated by 24–48 h. Two of the tasks were reinforcement learning tasks, where participants were instructed to learn the reward contingencies of abstract symbols. For both the reinforcement learning tasks and their different sessions, we used different sets of symbols such that for a participant, no symbols were repeated between tasks or sessions.

For *between-session* analysis of each task, we included all participants that completed both sessions for that task above chance level accuracy. Out of 150, we included 86 participants for the MSIT, 85 for the RB, 54 for the RL-SAT, and 54 for the RL-Rev. For analysis *between tasks*, we excluded 86 participants for not having completed all eight sessions, out of the remaining 64 participants, we further excluded 11 participants for performing below chance level accuracy on one of the eight sessions. The high drop-out rate in participants between sessions was likely due to the online, rather than in-lab, participation.

The experiment served as a pilot study for a functional magnetic resonance imaging (fMRI) experiment including five tasks. One of these tasks was the stop-signal task. The model architecture needed to account for the behavioral data of the stop-signal task is vastly different to the other tasks (Verbruggen et al., 2019; Matzke et al., 2017). Therefore, we chose not to include the stop-signal task in our analyses. In-depth methods including task background and design can be found in Appendix 1.

## Cognitive Modelling

### Modelling Overview

In the current study, we set out to test latent decision-making mechanisms that span different types of decision processes. To that end, we used four different types of decision-making tasks: (1) a reinforcement learning reversal learning task, in which learned reward-stimulus associations were switched following an acquisition phase (RL-Rev); (2) a cognitive control task that engaged both Flanker and Simon type interference (MSIT); (3) a working memory task in which participants were instructed to match the current stimulus to a reference stimulus (RB); and (4) a reinforcement learning tasks that interleaved speed emphasis and accuracy emphasis trials.

For each data set, we used a racing diffusion model (RDM; Zandbelt et al. , 2014; Tillman et al. , 2020). The RDM is an EAM that proposes a race between competing choices that each have separate accumulators. The first accumulator to reach the threshold determines the choice made, and the time taken to reach the threshold determines the response time along with the non-decision time. The RDM combines aspects of the DDM, as it posits within-trial noise in the accumulation process (Ratcliff & Smith, 2004), and the LBA, as it comprises separate accumulators for each choice (Brown & Heathcote, 2008). Here, we parameterized the drift rates as an evidence-independent urgency term ($V_0$) and an evidence-dependent difference term ($\delta$) (Fig. 1). The difference term can be interpreted as information processing ability, since it maps onto the ability of the decision-maker to discriminate the difference in evidence between the available choices. For the reinforcement learning tasks, we further included a sum term ($\Sigma$) that suggests that choosing between two more rewarding stimuli is faster than between two less rewarding stimuli. The sum term can therefore be interpreted as the sensitivity to reward of the decision-maker.

Each task also required specific changes to this overall model architecture that mapped model parameters to the task design. For the reinforcement learning tasks, the models we used were picked based on model comparisons described in Miletić et al. (2021). For the RB and MSIT, the models were picked based on formal model comparisons described in Appendix 2.

### Reinforcement Learning Tasks

We replicated the reinforcement learning speed-accuracy trade-off task (RL-SAT) and a reinforcement learning reversal task (RL-Rev) from Miletić et al. (2021), in which participants were tasked to learn the reward probabilities associated with different pairs of symbols. That experiment studied reciprocal influences of learning and decision-making, by integrating EAMs with reinforcement learning models (RL-EAMS; Fontanesi et al. , 2019a; Fontanesi et al. , 2019b; Frank et al. , 2015; Miletić et al. , 2021; Miletić et al. , 2020; Pedersen et al. , 2017; Sewell et al. , 2019). RL-EAMs propose that people make decisions by gradually integrating information of value representations associated with each available choice option. When enough evidence has been accumulated, they commit to a choice, and the associated feedback is used to update the value representations. In turn, these value representations drive the speed of evidence accumulation the next time the decision-maker is faced with the same choice.

In the previous study (Miletić et al., 2021), the authors relied on the delta learning rule to model the learning process:

1 $$\begin{aligned} Q_{t+1} = Q_t + \alpha (r - Q_t) \end{aligned}$$

This entails that the difference between reward *r* and the current expected value $Q_t$ is scaled by the learning rate $\alpha$ to determine how much the current value representation is updated to form the new expected value representation following feedback $Q_{t+1}$. These value representations drive the drift rate, the speed of evidence accumulation. That study showed that the choice of EAM in the RL-EAM greatly influenced the extent to which the RL-EAM could describe the data (Miletić et al., 2021). The authors concluded that the advantage framework in combination with a racing diffusion model (ARD) could best describe the learning-related increase in response accuracy and response speed. The advantage framework posits that the speed of evidence accumulation is a weighted sum of three components: first, a baseline, evidence-independent component, which can be interpreted as urgency (Miletić & van Maanen, 2019; Trueblood et al., 2021); second, the difference in perceived value between the available options; and third, the sum of the perceived values of all options (van Ravenzwaaij et al., 2019). Additionally, the evidence accumulation process is subject to Gaussian noise *W*, with standard deviation *s*, which was fixed to 1 to satisfy scaling constraints (Donkin et al., 2009; van Maanen & Miletić, 2020). When faced with two choices as in our instrumental learning tasks, the accumulators associated with each choice can thus be described as follows:

2 $$\begin{aligned}&dx_1 = [V_0 + \delta (Q_1 - Q_2) + \Sigma (Q_1 + Q_2)] + sW \nonumber \\&dx_2 = [V_0 + \delta (Q_2 - Q_1) + \Sigma (Q_1 + Q_2)] + sW \end{aligned}$$

Furthermore, non-decision time ($t_0$) and response caution (*B*) were also estimated.

The same model architecture, a combination of the advantage framework racing diffusion model with a reinforcement learning algorithm, was applied to both learning tasks. Below, we outline task-specific changes to the modelling architecture, if any, for the two reinforcement learning tasks.

### Reinforcement Learning Reversal Task

The reinforcement learning reversal task (RL-Rev) is an instrumental learning task in which the associated reward probabilities within a pair are switched roughly halfway through a block (Behrens et al., 2007; Costa et al., 2015). This tests the ability of the participant to update their representation of the most valuable stimulus within a stimulus pair. We used the above-described RL-ARD to account for the behavioral data of this task. The model predicts that following the reversal, the reward prediction errors will increase, which in turn will lead to updating of the *Q* values. The RL-ARD we used for the RL-Rev has six free parameters ($\alpha , V_{0}, \delta , \Sigma , B, t_0$).

### Reinforcement Learning Speed-Accuracy Trade-off Task

In the reinforcement learning speed-accuracy trade-off task (RL-SAT), participants complete an instrumental learning task in which they are instructed to emphasize response accuracy on half of the trials and response speed on the other half of the trials (Sewell et al., 2019). On these speed trials, participants also have less time to respond, forcing them to leverage speed for caution, which is referred to as the speed -accuracy trade-off (Ratcliff & Rouder, 1998; Bogacz et al., 2010). Similar to other recent papers (Rae et al., 2014; Arnold et al., 2015; Sewell et al., 2019; Heathcote & Love, 2012), Miletić et al. (2021) found that such SAT manipulations are best described by both drift rate adjustments (in this case separate urgency components, $V_{0, spd}$ $V_{0, acc}$), as well as threshold adjustments (different thresholds $b_{spd}$ and $b_{acc}$; Miletić et al. , 2021). In total, the model we used for the RL-SAT was a RL-ARD with eight free parameters ($\alpha , V_{0, spd}, V_{0, acc}, \delta , \Sigma , B_{spd}, B_{acc}, t_0$).

### Reference-Back Task

The reference-back task (RB) is a working memory task in which participants have to compare the current stimulus to a stimulus held in working memory (the reference) to make a binary “same” or “different” response. On comparison trials, the stimulus needs only to be compared to the reference stimulus held in working memory, whereas on reference trials, the stimulus also becomes the reference for subsequent trials (Rac-Lubashevsky & Kessler, 2016a, b). Stimuli were presented as either the letter “X” or “O,” encircled by a red frame for reference trials and a blue frame for comparison trials. Using the RDM, we can disentangle the cognitive costs of working memory updating (reference trials) from working memory comparison (comparison trials).

Previous studies showed that participants performed better when responding “same” compared to “different” and when there was a comparison rather than a reference trial. Furthermore, these studies also found a sequencing effect in that participants were faster when previous trial’s trial type (i.e., reference or comparison) was repeated compared to a switch in the trial type sequence (e.g., Rac-Lubashevsky & Kessler , 2016b; Boag et al. , 2021; Rac-Lubashevsky & Frank, 2021; Jongkees , 2020). In the current study, we also found that participants performed better following stimulus-identity (e.g., X or O) repetitions between trials compared to switches in stimulus identity. We aimed to construct the most parsimonious model that still accounted for the aforementioned effects and was similar in architecture as the RL-ARD described above. Therefore, instead of letting drift rates vary across response, trial type, trial type sequence, and stimulus sequence, we used a sensitivity parametrization, in which the drift rates for two options can be written as a combination of an urgency term and a difference term (similar to Strickland et al. , 2018), such that

3 $$\begin{aligned} v_{correct} = V_0 + \frac{\delta }{2}, \quad v_{error} = V_0 - \frac{\delta }{2} \end{aligned}$$

Thus, we can estimate some of the aforementioned effects as differences in $V_0$ (urgency) and some as differences in $\delta$ (processing ability), effectively reducing the number of parameters estimated. This parameterization is essentially a simplification of the advantage framework. To elaborate, the $V_0$ parameter in Eq. 3 captures both the urgency and the sum term of the advantage framework (Eq. 2), since these cannot be disentangled in a task where there are no inherent stimulus values. Analogously, the $\delta$ in the advantage framework weights the difference of stimulus values, whereas the $\delta$ in Eq. 3 captures the difference in evidence for each stimulus. Even though this parameterization is a simplification of the advantage framework, it still results in a range of models that capture the effects associated with the RB task. Since the above-described model was newly developed, we performed a model comparison study to test which combination of parameters could best account for our behavioral data (see Appendix 2). In the winning model, the evidence accumulation process for the correct and incorrect choice can be described as follows:

4 $$\begin{aligned}&dx_{correct} = [V_{0,type-trans} + \delta _{type, response}]dt + sW \nonumber \\&dx_{incorrect} = [V_{0,type-trans} - \delta _{type, response}]dt + sW \end{aligned}$$

This entails that the $\delta$ parameter varied depending on each combination of what would be the correct response (“same” or “different”; same choices are generally easier) and trial type (“reference” or “comparison”; comparison trials are generally easier). We also estimated an urgency term that differs for type transitions, e.g., repetitions in trial type or switches in trial type. Additionally, the winning model comprised a difference in threshold between repetitions in stimulus identity and switches in stimulus identity. In total, our RDM for the RB comprised ten parameters ($V_{0, type-trans_1} V_{0, type-trans_2}, \delta _{resp_1, type_1}, \delta _{resp_2, type 1},\delta _{resp_1, type_2}, \delta _{resp_2, type 2}, B_{stim-trans_1}, B_{stim-trans_2}, t_0$). We numbered the types of trials, transitions of trials, transitions of stimulus identity, and responses for brevity.

**Fig. 2:**
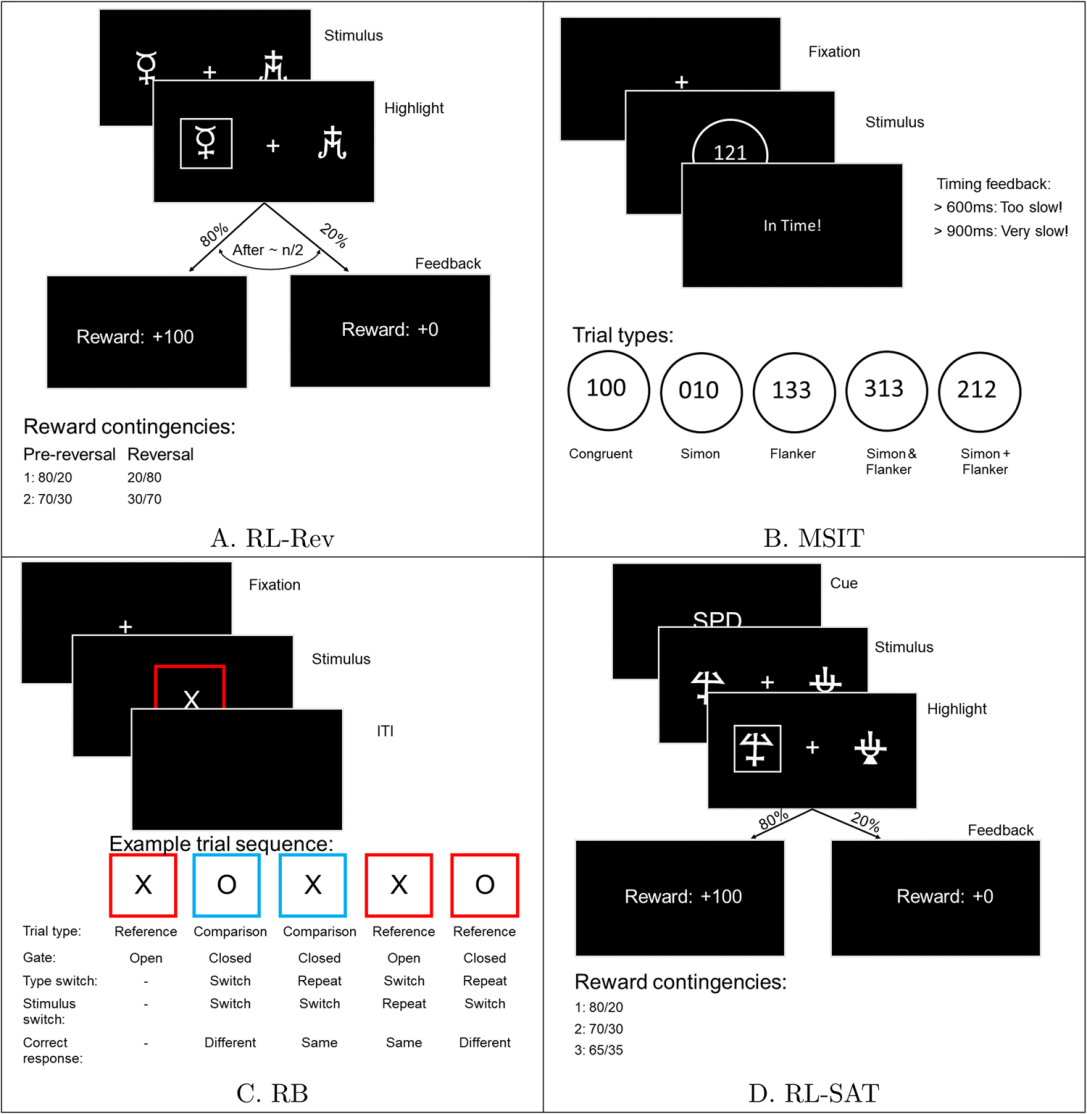
Visual descriptions of the four decision-making tasks. **A** The reversal learning task (RL-Rev). The reward contingencies are the reward probabilities associated with the two pairs of symbols presented per block. **B** The multi-source interference task (MSIT). **C** The reference-back (RB). **D** The reinforcement learning speed-accuracy trade-off

### Multi-source Interference Task

The multi-source interference task (MSIT; Bush et al. , 2003) combines two types of interference with the relevant (target) visual information, either from irrelevant location information (the Simon effect; Hommel , 2011) or irrelevant stimuli spatially adjacent to the target (the Flanker effect; Eriksen , 1995). In the current experiment, we use an adjusted version of the MSIT (Isherwood et al., 2022), where Flanker and Simon interference are presented both separately and combined in different types of trials (for example trials, see Fig. 2; for a more in-depth design description, see Appendix 1). Together, these different trial types allowed us to test the extent to which decision-making processes are influenced by Flanker or Simon interference and a possible combination of both. We rely on an EAM framework that introduces separate drift rates for both Flanker and Simon interference. In contrast to our other three decision-making tasks, participants always had three response options rather than two.

To the best of our knowledge, there have been no previous studies that used a model-based approach to analyze the MSIT. We therefore constructed a process-oriented model of decision-making that could best describe the observed behavioral effects. We hypothesized that the drift rate for each choice is jointly driven by an urgency component and the evidence supporting that choice. To quantify the evidence in support for each choice, we described the drift rate for choice A as a combination of possible Flanker support, Simon support, and target support (the correct response). Additionally, the evidence accumulation process is subject to Gaussian noise *W*, with standard deviation *s*, which again was fixed to 1 to satisfy scaling constraints. Consequently, the drift rates in our MSIT model can be described as follows:

5 $$\begin{aligned} dx = [V_0 + v_{Flank} + v_{Simon} + \delta ]dt + sW \end{aligned}$$

where $v_{Flank}$ and $v_{Simon}$ were 0 if there was no Flanker support or Simon effect respectively. Similarly, $\delta$ was only added to the accumulator that matched the correct response. Furthermore, we found that response time and accuracy were influenced by the position of the target, possibly due to left-to-right reading effects. We therefore modified the starting point of the accumulator corresponding to the target, based on the position of the target. We fixed $start_{pos_3}$ to 0 to set a base positional start point modifier to which the other positional modifiers were relative, to satisfy scaling constraints. In total, our MSIT model comprised eight parameters ($v_{Flank}, v_{Simon}, \delta , start_{pos_1}, start_{pos_2}, V_0, B, t_0$).

The above-described model was selected after model comparison against competing models (for details, see Appendix 2).

### Joint Model

For each task, we used the above-described models to first test to what extent the behavior of the participants on session 1 of a task is related to their behavior on session 2 of a task. To that end, we used a joint model of both sessions combined. These four *between-session* joint models had two components: the first component was the parameter set corresponding to the first session, and the second component was the parameter set corresponding to the second session.

We concatenated the parameters from the components into a single vector of parameters that we estimated per participant (random effects), and for each of those parameters, we also estimated a group-level multivariate normal distribution that describes the distribution of the parameters of all participants as a group. The multivariate normal distribution allowed us to estimate the covariances that existed between these group-level parameters (Turner et al., 2013; Gunawan et al., 2020):

6 $$\begin{aligned} p(\alpha , \mu , \Sigma \vert y) \sim p(y \vert \alpha ) p(\alpha \vert \mu , \Sigma ) p(\mu , \Sigma ) \end{aligned}$$

Here, *y* is the data of the two sessions, and $\alpha$ are the random effects that are concatenated across all sessions. The term $p(y \vert \alpha )$ constitutes the joint likelihood of the cognitive models across both sessions. Additionally, $\mu$ is the group-level mean across individuals, and $\Sigma$ is the group-level covariance matrix that captures the relationships between individuals across sessions. Here, $p(\alpha \vert \mu , \Sigma )$ describes the reciprocal relationship between the group-level distribution and the random effects, and $p(\mu , \Sigma )$ are the priors on the group-level parameters as described in Gunawan et al. (2020). We applied this covariance joint modelling architecture to construct four joint models of the different sessions for the different tasks.

Besides the between-session joint models, we similarly constructed a joint model that simultaneously estimated the parameters from all four task-specific models, which we will again refer to as components. In order to reduce the number of parameters we estimated, we pooled the data of both sessions for each task and estimated only one set of parameters per task. Nevertheless, if we combined the parameters estimated per participant from each task-specific model component, we would need to estimate 6 (RL-Rev) + 8 (RL-SAT) + 9 (RB) + 8 (MSIT) = 31 parameters per participant. If we then applied a multivariate normal distribution as a group-level distribution, it would result in 31 means + 31$\times$(31$-$1)/2 variances and covariances, thus a total of 496 group-level parameters. To reduce the number of estimated group-level parameters and thus increase the number of data points per parameter, we used hierarchical factor analysis rather than a hierarchical multivariate normal model (Innes et al., 2022). Such that rather than estimating $\Sigma$, we estimate a factor decomposition of $\Sigma$ at the group level that describes the relationships between individuals using latent factors:

7 $$\begin{aligned} \Sigma = \Lambda \Lambda ^T + \epsilon \end{aligned}$$

Here $\Lambda$ are the factor loadings and $\epsilon$ describes the diagonal residuals of the variances. The exact details of this decomposition are described in Innes et al. (2022). We can now write the full model as:

8 $$\begin{aligned} p(\alpha , \mu , \Lambda , \epsilon \vert y) \sim p(y \vert \alpha ) p(\alpha \vert \mu , \Lambda , \epsilon ) p(\mu , \Lambda , \epsilon ) \end{aligned}$$

Here, *y* is the data of the four tasks, and $\alpha$ are the random effects that are concatenated across all tasks. The term $p(y \vert \alpha )$ now constitutes the joint likelihood of the cognitive models across all tasks. As in Eq. 6, $\mu$ is the group-level mean across individuals; however, now, the relationships between individuals across sessions are described using $\Lambda$. Thus, $p(\alpha \vert \mu , \Lambda , \epsilon )$ describes the reciprocal relationship between the group-level distribution and the random effects, and $p(\mu , \Lambda , \epsilon )$ are the priors on the group-level parameters as described in Innes et al. (2022).

Besides the significant reduction in parameter space, the hierarchical factor structure also allows us to examine the latent factors for meaningful interpretation. To answer what number of factors optimally described our data, we estimated multiple joint factor models each with a different number of factors and interpreted the model with the highest marginal likelihood (the gold standard in Bayesian model comparison; Kass & Raftery, 1995), as estimated by the newly developed approach, importance sampling squared ($IS^2$; Innes et al. , 2022; Tran et al. , 2021). $IS^2$ uses importance sampling on both the individual level and the group level of the hierarchical model to obtain an estimate of the marginal likelihood. The importance samples can subsequently also be used to calculate Bayes factors (the ratio of two marginal likelihoods) and a bootstrapped standard error of these Bayes factors.

### Bayesian MCMC Sampling Using PMwG

All models were estimated using particle Metropolis within Gibbs sampling (PMwG; Gunawan et al. , 2020), which comprises three phases: burn-in, adaptation, and sampling. The group-level distributions, in our case the multivariate normal or the factor decomposition of it, is described using Gibbs sampling (George & Mcculloch, 1993). For the group-level mean parameters, we used a multivariate normal prior with variance 1 and covariance 0. The prior mean for the group-level means was set to 2 for threshold (*B*) parameters, 2 for processing ability ($\delta$) parameters, 1 for urgency ($V_0$) parameters, 0.2 for non-decision time (*t*0) parameters, and 0 for all other parameters. For the prior on the group-level covariance and factor structure, we relied on the default priors described in Gunawan et al. (2020) and Innes et al. (2022) respectively.

The parameters at the individual level are estimated using particle Metropolis-Hastings sampling (Chib & Greenberg, 1995), which uses different proposal distributions in combination with importance sampling. In the burn-in phase, these proposals are drawn jointly from the group-level distribution at that Markov chain Monte Carlo (MCMC) iteration and a multivariate normal distribution centered on the previous set of random effects of that individual in the MCMC chain. Following burn-in, the parameters have converged towards their posterior; therefore, in the adaptation stage, we draw samples that approximate the posterior distribution. We subsequently use these samples to create a distribution that mimics the posterior to efficiently draw proposals from. In the sampling stage, this efficient distribution, together with the group-level distribution and the distribution centered on the previous set of parameters of that individual, is used to draw proposals for the particle Metropolis-Hastings step for each individual (Gunawan et al., 2020).

## Results

Participants each completed four different decision-making tasks twice. We analyzed the data using different joint models that explicitly estimated the relationships *between sessions* or *between tasks* in the architecture of the model. Of the task-specific models that formed the components of the joint models, the models of the RL-Rev and RL-SAT were selected based on model comparisons described in Miletić et al. (2021). The models of the MSIT and RB were newly developed and selected after a model comparison study (see “Materials and Methods”).

### Between Sessions

For each task, we used a joint model of both sessions to test to what extent the behavior was related between the two sessions. These four *between-session* joint models had two components: the first component comprised the EAM parameters corresponding to the first session, and the second component comprised the EAM parameters corresponding to the second session. Our *between-session* joint models relied on a multivariate normal distribution at the group-level to account for the relationships between the parameters. We included data from 54 participants in the *between-sessions* joint model of the RL-Rev, 86 participants for the MSIT, 85 for the RB, and 54 for the RL-SAT (see “Materials and Methods”). In Table 1, we report the credible intervals of the group-level mean parameters for sessions one and two as well as the response times and accuracy across participants. Across tasks, we note that information processing ability ($\delta$) and urgency ($V_0$) were higher in session two compared to session one, whereas response caution (*B*) was lower. This is consistent with the finding that response times decreased in session two whereas response accuracy was more or less stable.

**Table 1:** 95% credible intervals of the group-level means, response times across participants, and accuracy across participants for sessions 1 and 2 for the different tasks

	Session 1			Session 2		
Variables	2.5%	50%	97.5%	2.5%	50%	97.5%
**RL-Rev**						
$\delta$	1.32	1.50	1.68	1.38	1.58	1.78
*B*	1.53	1.63	1.74	1.33	1.42	1.52
$t_0$	0.11	0.13	0.15	0.12	0.14	0.17
$V_0$	1.91	2.08	2.26	2.04	2.21	2.38
$\alpha$	0.15	0.18	0.20	0.16	0.19	0.22
$\Sigma$	0.34	0.48	0.63	0.33	0.49	0.65
*RT*	0.49	0.67	0.79	0.38	0.55	0.65
*Acc*	0.55	0.64	0.81	0.57	0.65	0.76
**MSIT**						
$t_0$	0.19	0.21	0.23	0.20	0.22	0.24
$v_{Fl}$	1.02	1.13	1.25	1.08	1.22	1.35
$v_{Si}$	0.99	1.13	1.26	1.02	1.17	1.32
$strt_{p_1}$	$-$0.02	0.04	0.09	0.00	0.06	0.11
$strt_{p_2}$	0.05	0.11	0.17	0.01	0.06	0.12
$\delta$	2.40	2.60	2.82	2.68	2.90	3.12
$V_0$	0.48	0.68	0.88	0.75	0.98	1.28
*B*	1.71	1.83	1.97	1.63	1.75	1.91
*RT*	0.54	0.62	0.82	0.48	0.55	0.71
*Acc*	0.71	0.84	0.93	0.71	0.85	0.93
**RB**						
$t_0$	0.10	0.12	0.14	0.12	0.14	0.16
$V_{0_{tt_1}}$	1.84	1.97	2.11	2.14	2.32	2.52
$V_{0_{tt_2}}$	1.66	1.78	1.90	1.97	2.13	2.30
$\delta _{tr_1}$	2.30	2.57	2.83	2.49	2.81	3.11
$\delta _{tr_2}$	2.35	2.60	2.84	2.65	3.00	3.36
$\delta _{tr_3}$	1.95	2.19	2.42	2.28	2.57	2.87
$\delta _{tr_4}$	1.42	1.63	1.87	1.72	1.98	2.24
$B_{st_{1}}$	1.56	1.68	1.80	1.38	1.50	1.63
$B_{st_{2}}$	1.77	1.90	2.04	1.56	1.69	1.81
*RT*	0.59	0.73	1.45	0.44	0.59	1.01
*Acc*	0.66	0.92	0.98	0.84	0.92	0.97
**RL-SAT**						
$t_0$	0.09	0.12	0.14	0.11	0.14	0.17
$V_{0_{c_1}}$	2.83	3.06	3.25	3.21	3.53	3.81
$V_{0_{c_2}}$	2.43	2.74	3.01	2.71	3.11	3.46
$B_{c_{1}}$	1.65	1.79	1.92	1.48	1.71	1.91
$B_{c_{2}}$	1.77	1.99	2.21	1.55	1.80	2.01
$\Sigma$	0.45	0.66	0.97	0.12	0.44	0.76
$\delta$	2.03	2.47	3.18	2.12	2.49	3.21
$\alpha$	0.04	0.07	0.10	0.05	0.08	0.11
*RT*	0.43	0.57	0.68	0.41	0.53	0.61
*Acc*	0.60	0.68	0.86	0.56	0.65	0.76

**Table 2:** 95% credible intervals of the correlations between the same parameters of sessions 1 and 2 for the different tasks

	Correlation estimates		
Variable	2.5%	50%	97.5%
**RL-Rev**			
$t_0$	$-$0.14	0.16	0.42
$V_0$	**0**.**11**	**0**.**41**	**0**.**66**
*B*	$-$0.21	0.11	0.41
$\delta$	-.40	$-$0.15	0.12
$\alpha$	-.46	$-$0.16	0.19
$\Sigma$	-.38	$-$0.08	0.26
**MSIT**			
$t_0$	**0**.**06**	**0**.**39**	**0**.**63**
$V_{Fl}$	**0**.**09**	**0**.**36**	**0**.**60**
$V_{Si}$	**0**.**33**	**0**.**55**	**0**.**73**
$strt_{p_1}$	**0**.**15**	**0**.**44**	**0**.**66**
$strt_{p_2}$	**0**.**14**	**0**.**40**	**0**.**63**
$\delta$	**0**.**33**	**0**.**55**	**0**.**72**
$V_0$	$-$0.06	0.25	0.55
*B*	$-$0.14	0.24	0.54
**RB**			
$t_0$	**0**.**06**	**0**.**37**	**0**.**62**
$V_{0_{tt_1}}$	**0**.**20**	**0**.**48**	**0**.**69**
$V_{0_{tt_2}}$	**0**.**17**	**0**.**42**	**0**.**65**
$\delta _{tr_1}$	**0**.**18**	**0**.**48**	**0**.**69**
$\delta _{tr_2}$	**0**.**29**	**0**.**55**	**0**.**74**
$\delta _{tr_3}$	**0**.**27**	**0**.**55**	**0**.**72**
$\delta _{tr_4}$	**0**.**20**	**0**.**47**	**0**.**68**
$B_{st_{1}}$	**0**.**23**	**0**.**53**	**0**.**75**
$B_{st_{2}}$	**0**.**29**	**0**.**56**	**0**.**75**
**RL-SAT**			
$t_0$	**0**.**19**	**0**.**46**	**0**.**66**
$V_{0_{c_1}}$	**0**.**26**	**0**.**55**	**0**.**76**
$V_{0_{c_2}}$	**0**.**42**	**0**.**64**	**0**.**79**
$B_{c_{1}}$	**0**.**52**	**0**.**72**	**0**.**86**
$B_{c_{2}}$	**0**.**58**	**0**.**76**	**0**.**87**
$\Sigma$	-.15	0.20	0.50
$\delta$	**0**.**13**	**0**.**48**	**0**.**72**
$\alpha$	**0**.**34**	**0**.**60**	**0**.**78**

Correlations with credible intervals not containing 0 are marked in bold

Furthermore, we translated the covariances of the multivariate normal group-level into correlations. For all tasks, we constructed credible intervals from the marginal posterior distributions, for the correlations between parameters of the different sessions that mapped onto the same cognitive construct (e.g., the correlation between $t_0$ of session 1 and $t_0$ of session 2). We found that these credible intervals spanned only positive values for most of the parameters of the RL-SAT, RB, and MSIT (see Table 2). These positive correlations indicate that participants who, for example, showed high response caution in session 1 also showed high response caution in session 2.

**Fig. 3:**
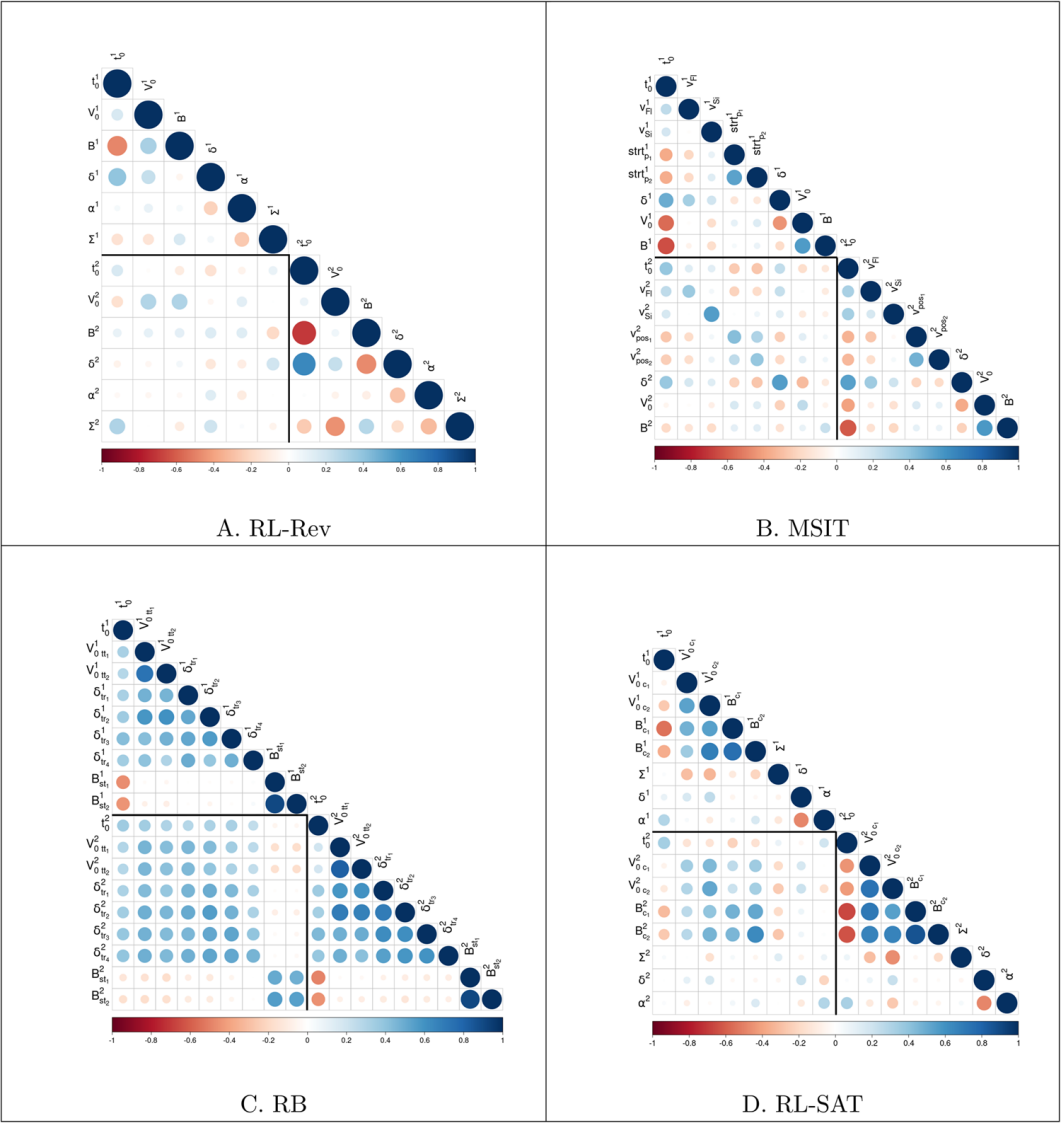
Mean parameter correlations of the joint models of the two sessions of the different tasks. The larger the circles, the larger the absolute size of the correlations. The darker the red, the more negative the correlations, and the darker the blue, the more positive the correlations. The black lines within each figure delineate the within and between-session correlations

We also plotted the correlation matrices for the different tasks (Fig. 3). The within-session correlations are symmetrical along the diagonal ($\rho _{v_{ses1},b_{ses1}} = \rho _{b_{ses1},v_{ses1}}$), and we therefore only plot the values below the diagonal. The between-session correlations, which are then found in the lower square of the triangular plots, are, however, not symmetrical (e.g. $\rho _{v_{ses1},b_{ses2}} \ne \rho _{b_{ses1},v_{ses2}}$). We found that again for the RL-SAT, RB, and MSIT, the visual pattern of correlations observed between the parameters within the same session is replicated between the parameters of the different sessions, albeit weaker in strength (see Fig. 3). This entails that if, for example, information processing ability and response caution were correlated constructs within a session, they were also correlated between sessions.

For the RL-Rev, we found that the 95% credible intervals for the correlations between the parameters that mapped onto the same cognitive construct did contain 0 for most parameters (Table 2), which indicates that the correlations between sessions for the RL-Rev were inconclusive. Furthermore, the same visual pattern of correlations within session was not found between the two sessions, which again highlights that there was lower similarity between the behavior in session 1 and session 2 of the RL-Rev compared to the other three tasks.

**Fig. 4:**
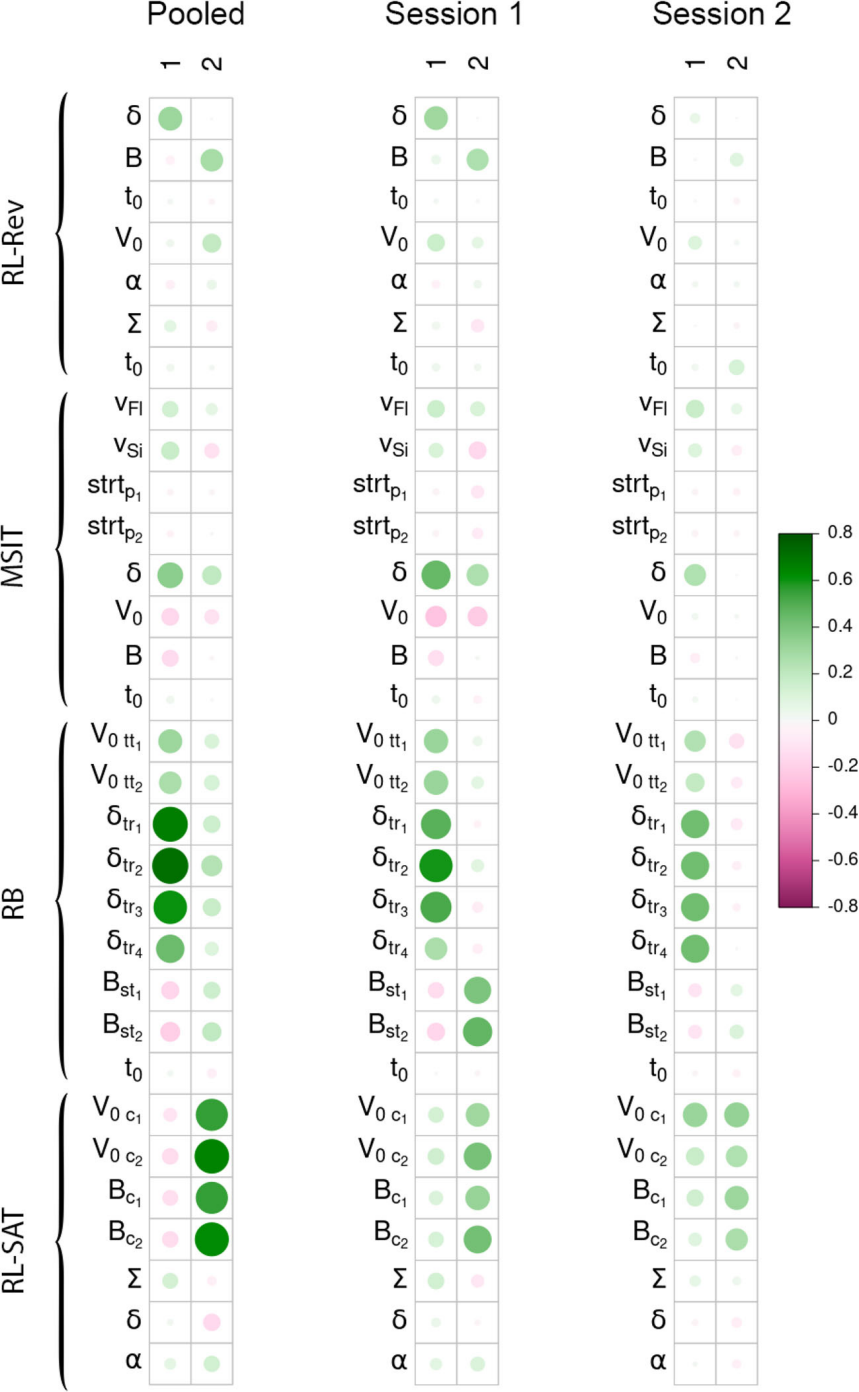
Mean factor loadings of the winning two-factor joint model of the four decision-making tasks. The larger the circles, the larger the absolute size of the factor loadings. The darker the pink, the more negative the correlations, and the darker the green, the more positive the correlations

### Between Tasks

We constructed a joint model that simultaneously estimated the parameters from all four task-specific models, which we will again refer to as components. In order to reduce the number of parameters we estimated, we pooled the data of both sessions for each task and estimated only one set of parameters per task. To further reduce the number of estimated group-level parameters and thus increase the number of data points per parameter, we used hierarchical factor analysis (see “Materials and Methods”; Innes et al. , 2022). Furthermore, we can also examine the latent factors given by the hierarchical factor model for meaningful interpretation. Rather than inspecting separate correlation estimates, these latent factors can be interpreted across tasks.

To determine which number of factors best described the relationships between the parameters of the joint model, we estimated the marginal likelihood for one, two, and three-factor models using a recently developed implementation of $IS^2$ for hierarchical factor models, which relies on importance sampling to obtain estimates of the Bayes factors (Innes et al., 2022; Tran et al., 2021). These importance samples can subsequently also be used to obtain standard errors of the Bayes factor estimates. We found that the group-level relationships were best described with two factors, standard errors in brackets [$BF_{2-1} = 35.97 (SE = 2.27), BF_{2-3} = 57.12 (SE = 2.90)$]. We did not estimate a four-factor model, since the increased complexity of a three-factor model was already not preferred over the two-factor model (Innes et al., 2022). On an exploratory basis, we also investigated this two-factor model applied to the first and second sessions of the data separately.

**Table 3:** 95% credible intervals of the factor loadings of the winning two-factor joint model of the four decision-making tasks with the two sessions pooled

	Factor 1 loadings			Factor 2 loadings		
Variable	2.5%	50%	97.5%	2.5%	50%	97.5%
**RL-Rev**						
$\delta$	**0**.**23**	**0**.**30**	**0**.**40**	0	0	0
*B*	$-$0.18	$-$0.05	0.11	**0**.**22**	**0**.**28**	**0**.**37**
$t_0$	$-$0.03	0.02	0.7	$-$0.07	$-$0.02	0.03
$V_0$	$-$0.14	0.015	0.20	**0**.**02**	**0**.**18**	**0**.**36**
$\alpha$	$-$0.14	$-$0.05	0.05	$-$0.05	0.05	0.16
$\Sigma$	$-$0.05	$-$0.08	0.21	$-$0.19	$-$0.06	0.07
**MSIT**						
$t_0$	$-$0.02	0.03	0.08	$-$0.04	0.02	0.07
$v_{Fl}$	**0**.**01**	**0**.**18**	**0**.**34**	$-$0.08	0.08	0.23
$v_{Si}$	**0**.**01**	**0**.**18**	**0**.**34**	$-$0.29	$-$0.12	0.04
$strt_{p_1}$	$-$0.08	$-$0.01	0.05	$-$0.09	$-$0.01	0.05
$strt_{p_2}$	$-$0.07	$-$0.02	0.04	$-$0.08	$-$0.01	0.06
$\delta$	**0**.**08**	**0**.**34**	**0**.**61**	$-$0.08	0.21	0.52
$V_0$	$-$0.38	$-$0.17	0.03	$-$0.34	$-$0.14	0.06
*B*	$-$**0.25**	$-$**0.14**	$-$**0.04**	$-$0.17	$-$0.03	0.11
**RB**						
$t_0$	$-$0.02	0.03	0.08	$-$0.05	0.00	0.05
$V_{0_{tt_1}}$	**0**.**16**	**0**.**28**	**0**.**40**	$-$0.07	0.12	0.29
$V_{0_{tt_2}}$	**0**.**13**	**0**.**25**	**0**.**36**	$-$0.03	0.14	0.28
$\delta _{tr_1}$	**0**.**45**	**0**.**66**	**0**.**89**	$-$0.22	0.22	0.51
$\delta _{tr_2}$	**0**.**42**	**0**.**66**	**0**.**91**	$-$0.10	0.28	0.61
$\delta _{tr_3}$	**0**.**42**	**0**.**63**	**0**.**89**	$-$0.18	0.25	0.53
$\delta _{tr_4}$	**0**.**30**	**0**.**45**	**0**.**63**	$-$0.18	0.12	0.36
$B_{st_{1}}$	$-$**0.31**	$-$**0.19**	$-$**0.06**	$-$0.01	0.13	0.27
$B_{st_{2}}$	$-$**0.37**	$-$**0.22**	$-$**0.07**	0.02	0.17	0.33
**RL-SAT**						
$t_0$	$-$0.04	0.02	0.07	$-$0.10	$-$0.05	0.00
$V_{0_{c_1}}$	$-$0.44	$-$0.13	0.30	**0**.**39**	**0**.**61**	**0**.**90**
$V_{0_{c_2}}$	$-$0.55	$-$0.18	0.30	**0**.**42**	**0**.**68**	**0**.**94**
$B_{c_{1}}$	$-$0.43	$-$0.16	0.20	**0**.**43**	**0**.**60**	**0**.**81**
$B_{c_{2}}$	$-$0.48	$-$0.17	0.21	**0**.**44**	**0**.**65**	**0**.**86**
$\Sigma$	**0**.**01**	**0**.**15**	**0**.**31**	$-$0.19	$-$0.01	0.16
$\delta$	$-$0.14	0.06	0.26	$-$0.32	$-$0.12	0.07
$\alpha$	$-$0.09	0.05	0.20	$-$0.02	0.11	0.27

Loadings with credible intervals outside of 0 are marked in bold

The mean group-level factor loadings of our two-factor model of the pooled data and the two sessions are plotted in Fig. 4. The 95% credible intervals and the median for all loadings of the pooled data are presented in Table 3. We found that all information discrimination ability ($\delta$) parameters, except for the RL-SAT, loaded positively onto the first factor. Furthermore, other evidence accumulation parameters of the MSIT and the RB also loaded positively onto the first factor. Thus, our first factor appeared to capture elements of the ability to discriminate information and of general evidence accumulation across tasks. Note that we also found weak negative loadings on the threshold parameters on the first factor for the MSIT and RB task, which suggests that participants who were good at the tasks also set lower thresholds for these tasks, as they could discriminate evidence more quickly. Furthermore, we found that parameters that related to threshold (*B*) or urgency ($V_0$) from the reinforcement learning tasks loaded highly on the second factor. Thus, our second factor was related to time management in the reinforcement learning tasks. Note that the first parameter ($\delta$ of the RL-Rev) on the second factor was fixed to 0 to satisfy estimation constraints (Innes et al., 2022; Ghosh & Dunson, 2009).

These findings were mostly consistent with the factor models applied to the separate sessions. However, for the first session, we found that thresholds in the RB task also loaded highly on the second factor. Furthermore, for the data of the second session, we found that the parameters of the RL-Rev did not have strong loadings on either factor.

## Discussion

In this study, we investigated to what extent decision-making behavior is related between different types of decisions. To that end, we tested participants on four different decision-making tasks: a reversal learning task (RL-Rev; Behrens et al. , 2007; Costa et al. , 2015), a reinforcement learning speed-accuracy trade-off task (RL-SAT; Sewell et al. , 2019; Miletić et al. , 2021), a working memory task called the reference-back task (RB; Rac-Lubashevsky & Kessler, 2016a; Rac-Lubashevsky & Kessler , 2016b), and a cognitive control task, called the multi-source interference task (MSIT; Bush et al. , 2003; Isherwood et al. , 2022). We relied on evidence accumulation models (EAMs) tailored to each task to account for both modalities of decision-making behavior, responses, and response times. Furthermore, EAMs facilitate the interpretation of the behavior in terms of latent cognitive constructs (the parameters in the EAMs) underlying the data (Donkin & Brown, 2018; Ratcliff et al., 2016).

For the two learning tasks, we relied on previously developed and tested models that capture the interplay between learning and decision-making (Miletić et al., 2021). However, for the RB and MSIT, we developed two novel modelling approaches that provided a parsimonious account of the different experimental effects in the respective designs.

To test relationships between the parameters of our different EAMs, we employed the joint modelling framework, where relationships between parameters are measured with less attenuation compared to standard approaches, using a hierarchical model that explicitly estimates the relationships between the parameters as an integral part of the model (Turner et al., 2013, 2017; Wall et al., 2021; Matzke et al., 2017). First, for each task, we constructed a *between-session* joint model of the two sessions participants had completed to test to what extent the cognitive constructs, as proposed by EAMs, were consistent between different time points. Then, we constructed a *between-task* joint model to investigate to what extent these constructs were related between different decision-making domains.

### Between Sessions

The *between-session* joint models of the RL-SAT, RB, and MSIT showed high correlations between the same latent constructs of the first and second sessions. Furthermore, parameters that were correlated within session for these three tasks were predominantly also correlated between the two sessions, which indicates that strategic components of the decision-making process were consistent between the two sessions. For example, we found that participants who showed higher non-decision times in session one not only showed lower response caution within that same session to compensate for the time taken, but also in session two. The correlations across sessions between the same cognitive constructs and between strategic components together provide evidence that EAMs capture a high degree of similarity in behavior of the RL-SAT, RB, and MSIT.

In contrast to the three other tasks, for the RL-Rev, we did not find these patterns of high between-session correlations between the same constructs or strategic components. In the RL-Rev, participants learned to identify to most rewarding stimulus within a pair of stimuli; however, roughly halfway through each block, the least rewarding stimulus of the pair became the most rewarding and vice versa (Behrens et al., 2007; Costa et al., 2015). This reversal is not explicitly instructed to the participants. Thus, the dissimilarity between the two sessions is potentially caused by the participants slightly adjusting their behavior in the second session in anticipation of the reversal, and the reversal learning task might not be suitable for between-session aims.

### Between Tasks

Our primary interest was to test to what extent the latent cognitive constructs as proposed by EAMs were related between different decision-making domains. To that end, we constructed a joint hierarchical factor model that captured the relationships between the parameters of four different decision-making tasks using latent factors (Innes et al., 2022). We found that the relationships between the parameters of the joint model were best described using two factors.

Our first factor indicated that an individuals’ ability to discriminate information quickly was related between the different types of decisions they faced in three of our four tasks. Furthermore, we also found that for the cognitive control task and working memory task, individuals who were good at information processing subsequently also demonstrated less response caution, potentially because of their ability to discriminate the correct from incorrect choices more quickly. Information processing ability in the reinforcement learning speed-accuracy trade-off task did not load highly on the first factor, but rather the sensitivity to the overall available reward. Potentially, the speed pressure of the speed emphasis trials shifted the decision-making mechanics to be more sensitive to the overall reward.

The second factor in our hierarchical factor model provided support that strategic components were not fully consistent across all tasks. Namely, the second factor indicated that participants who demonstrated a stronger sense of urgency, an evidence-independent part of the evidence accumulation process (Miletić & van Maanen, 2019), also showed higher response caution, but only in the two reinforcement learning tasks. The concept of urgency as defined in our models has comparable effects on predicted behavior as the concept of collapsing bounds in EAMs (Miletić & van Maanen, 2019; Cisek et al., 2009; Thura & Cisek, 2016; Hawkins et al., 2015). Thus, presumably participants that exhibited a stronger sense of urgency had to compensate this urgency by setting higher thresholds. Note that urgency and thresholds have unique contributions to the decision-process. High urgency paired with high response caution yields lower accuracy with increasing response times compared to the combination of low urgency and low response caution (Trueblood et al., 2021; Miletić & van Maanen, 2019). Thus, we found evidence that a strategic component of time management was related across the two reinforcement learning tasks, but not the other two decision-making tasks.

Furthermore, on an exploratory basis, we also separately studied the first and second sessions of all tasks in two separate factor models. We found similar results for both sessions pooled together. However, we found that for the data of the second session, the behavior on the reversal learning task did not show any consistencies with the behavior of the other tasks. This again corroborates our findings on the between-session analysis of the reversal learning task, that behavior changed between the first and second sessions, potentially because of an anticipated reversal.

The results of our analyses are in keeping with earlier work showing that the drift rate, which maps onto the latent cognitive process of information processing ability or cognitive efficiency, is mostly consistent across decision-making domains (Lerche et al., 2020; Schubert et al., 2016; Weigard et al., 2021; Schmiedek et al., 2007). The current work builds on the aforementioned approaches, first by taking into account more complex decision-making tasks from various domains. Second, by employing the joint modelling framework, specifically a joint hierarchical factor analysis (Innes et al., 2022), that explicitly takes into account relationships between the parameters of our models to reduce attenuation of the estimated relationships and to therefore obtain more accurate estimates.

Although we did find information processing ability to be mostly related between different types of decisions across our tasks, we found strategic differences between the two reinforcement learning tasks and the other tasks. A limitation of the current work is that we cannot rule out whether these inconsistencies are caused by the dissimilarity in behavior between these tasks or by the dissimilarity in the modelling architecture, since we could not apply the exact same model to all four tasks. Although we attempted to keep the task-specific models as similar in architecture as possible, the inherent differences between the different tasks also required distinct modelling choices. Within the definition of an EAM, psychologists attribute meaning to parameters of the model. However, with complex tasks, we could only keep these parameters as similar in mathematical definition as possible, and we were limited by the differences in the designs of the tasks.

Nevertheless, this limitation holds for any analysis aimed at comparing relationships of cognitive processes between different types of decisions. Even in studies that performed subtraction analysis of response times, these subtractions are mapped onto inferred latent states, which can show even weaker consistency compared to the latent constructs as proposed by EAMs (Weigard et al., 2021; Price et al., 2019).

The current study highlights that interpretation of inferred latent states between decision-making tasks must be done with proper caution, since what can be referred to as urgency in one task could map onto a different cognitive process in another. Possibly, to take response caution, for example, people could employ different response caution mechanisms for different types of decisions, which is why we did not find strong relationships between response caution across all four tasks. Alternatively, people do employ the same response caution mechanism across different types of decisions, but our models failed to isolate this mechanism in our response caution parameters, and different cognitive processes partially mapped onto our response caution parameters. The absence of a one-to-one mapping of processes to parameters is of course to be expected, since models are inherently simplifications (Marr & Poggio, 1977; Guest & Martin, 2021). However, it becomes problematic when the processes-to-parameter mapping differs between different models, which could explain the inconsistencies in relationships estimated between our parameters. Future work could structurally explore to what extent the cognitive constructs proposed by EAMs differ between tasks with varying degrees of similarity, both in terms of modelling architecture and design.

In summary, in the current work, we found that an individual’s ability to process information quickly and accurately was related between the different types of decisions they faced in our four tasks. Furthermore, three of our four tasks showed high consistency in the proposed cognitive processes across individuals, and the fourth task had an element of surprise that was potentially lost in the second session. Because of the flexibility of the proposed framework, our methods can be easily extended to include models of neural data to study cortical and sub-cortical networks involved in decision-making. Therefore, we believe that the joint modelling framework should be utilized to study cognitive processes at the core of decision-making.

## Figures and Tables

**Fig. 5:**
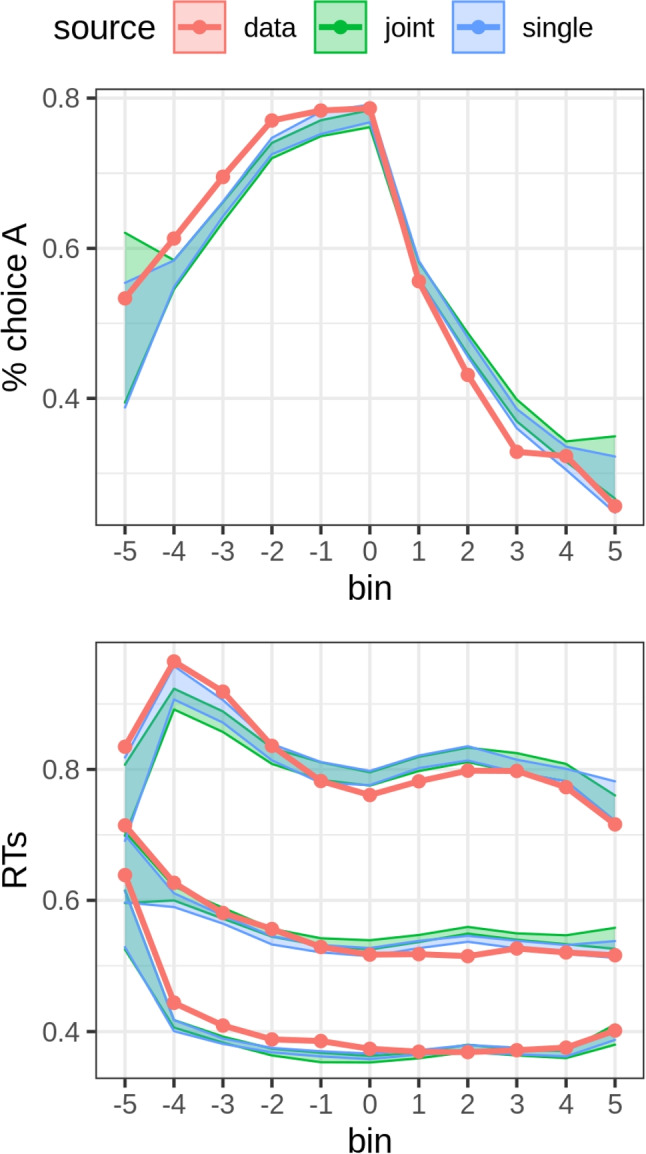
Posterior predictives for the reversal learning task. Top row: choice proportions over trials, with choice option A defined as the high-probability choice before the reversal in reward contingencies. Bottom row: 10th, 50th, and 90th RT percentiles. Shaded areas correspond to the 95% credible interval of the posterior predictive distributions of the joint model and the model fit individually

**Fig. 6:**
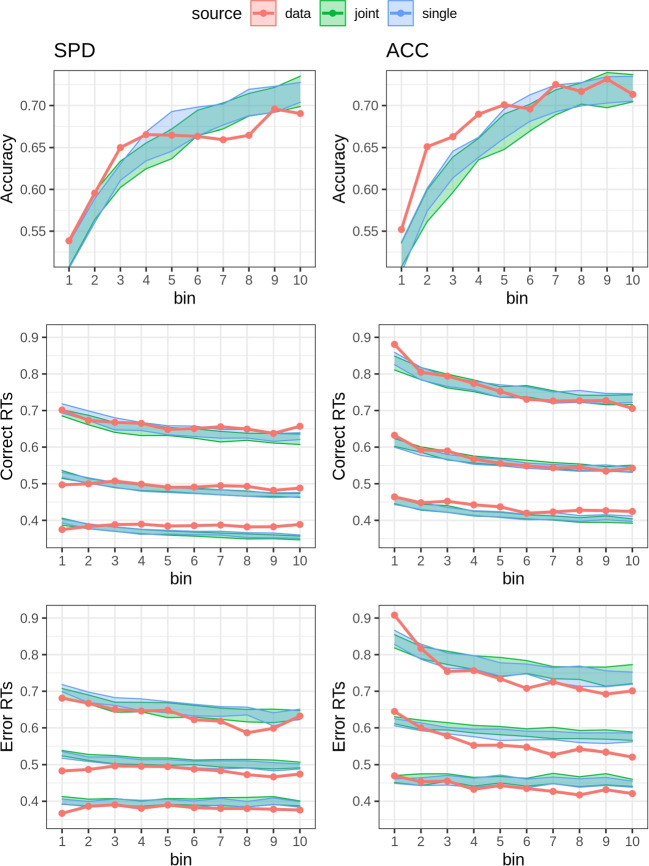
Posterior predictives for the reinforcement learning speed-accuracy trade-off task. Left: the data for all the trials where the speed cue was presented. Right: the trials where the accuracy cue was presented. The top row depicts accuracy over trial bins. The middle and bottom rows show 10th, 50th, and 90th RT percentiles for the correct (middle row) and error (bottom row) response over trial bins. Shaded areas in the middle and right column correspond to the 95% credible interval of the posterior predictive distribution

**Fig. 7:**
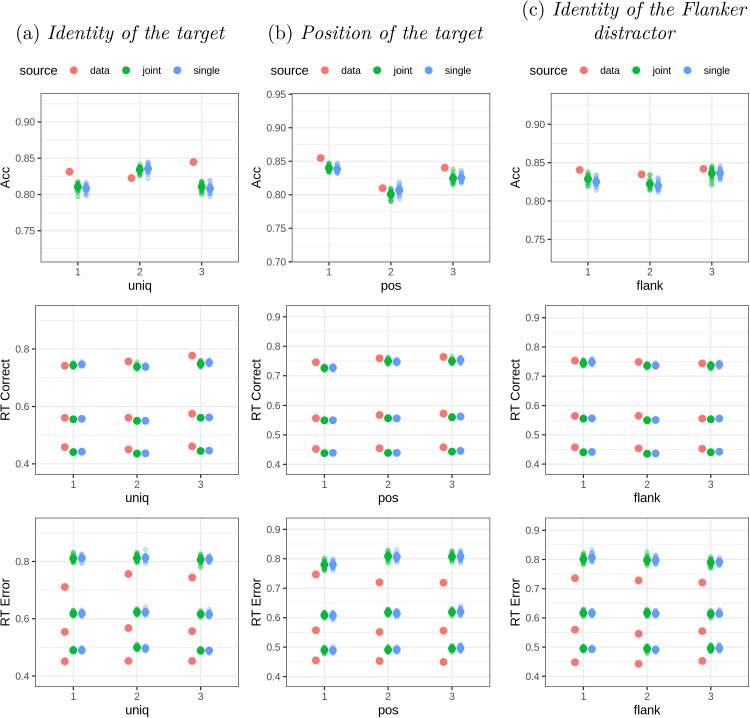
Posterior predictives for the multi-source interference task. **a** The data aggregated across the three different target identities. **b** The data aggregated across the three different positions where the target could be presented, which is the Simon distractor if it does not have the same identity as the target. **c** The data aggregated across the different possible identities of the Flanker distractors. The top row depicts accuracy over trial bins. The middle and bottom rows show 10th, 50th, and 90th RT percentiles for the correct (middle row) and error (bottom row) response over trial bins. Shaded areas in the middle and right column correspond to the 95% credible interval of the posterior predictive distribution

**Fig. 8:**
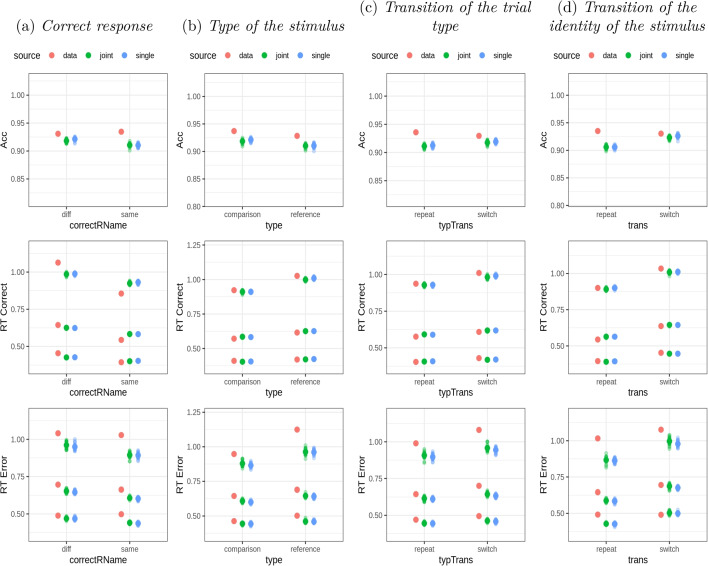
Posterior predictives for the reference-back task. **a** The data aggregated across the two different correct possible responses. **b** The data aggregated across the two different trial types. **c** The data aggregated across the different transitions in trial type compared to the trial type of the previous trial. **d** The data aggregated across the different transitions in stimulus identity compared to the stimulus identity of the previous trial. The top row depicts accuracy over trial bins. The middle and bottom rows show 10th, 50th, and 90th RT percentiles for the correct (middle row) and error (bottom row) response over trial bins. Shaded areas in the middle and right column correspond to the 95% credible interval of the posterior predictive distribution

## Data Availability

All code, data, and MCMC samples are publicly available on OSF (https://osf.io/c3d75/).

## Appendix 1. Design

### Reinforcement learning tasks

Two of the four decision-making tasks were replications of probabilistic instrumental learning tasks (Frank et al., 2004) described in Miletić et al. (2021). Here, we describe the general paradigm, after which we will highlight the differences between the two tasks. On each trial, participants chose between two abstract symbols that were both associated with a fixed probability of returning reward when chosen. Within that pair of stimuli, one of the choice options always had a higher chance of returning reward than the other. The aim for the participant was to maximize returns, by learning through trial and error which of the two symbols was associated with a higher chance of returning reward. If a choice was rewarded, the reward was equal to 100 points.

After each choice, participants received feedback consisting of two components: an outcome and a reward. The outcome refers to the outcome of the probabilistic gamble, whereas the reward refers to the number of points the participant actually received. If the participant responded in time, the reward was equal to the outcome.

#### Reinforcement learning reversal learning task

In the reinforcement learning reversal task (RL-Rev), we used the above-described paradigm with the following settings. Participants completed two sessions, each with two blocks and 128 trials per block. Two pairs of symbols with associated reward probabilities 8/.2 and.7/.3 were presented in a block, randomly interleaved. Between trials 61 and 68 (sampled from a uniform distribution) of each block, the associated reward probabilities within a pair were switched, such that what used to be the most rewarding stimulus within a pair now became the least rewarding of the two stimuli. No symbols were repeated between the blocks. Participants were not informed of the reversal in the instructions of the experiment. On each trial, the pair of symbols was presented until a participant made a response, but with a maximum of 2000 ms. Following stimulus presentation, the choice was highlighted for 500 ms after which feedback was presented for 750 ms. One session of the RL-Rev was always paired with a session of the reference-back task. The order of the tasks within session and between the two sessions was counterbalanced between participants.

On each trial, the pair of symbols was presented until a participant made a response, but with a maximum of 2000 ms. Following stimulus presentation, the choice was highlighted for 500 ms after which feedback was presented for 750 ms.

One session of the RL-Rev was always paired with a session of the reference-back task. The order of the tasks within session and between the two sessions was counterbalanced between participants.

#### Reinforcement learning speed-accuracy trade-off task

In order to manipulate the speed-accuracy trade-off, a cue and a deadline manipulation were added to the reinforcement learning paradigm (Frank et al., 2004). Prior to each trial, a cue instructed participants to emphasize response speed (“SPD”) or accuracy (“ACC”). Participants did not earn a reward in speed trials if they responded slower than 600 ms. Speed and accuracy trials were randomly interleaved.

Participants completed two sessions with each having 324 trials with 108 trials per block. In each block, three different pairs of symbols were each presented 36 times, with associated reward probabilities 0.8/0.2, 0.7/0.3, and 0.6/0.4. No symbols were repeated between the blocks. On each trial, the cue was presented on screen for 1500 ms, after which the pair of symbols was presented until a participant made a response, but with a maximum of 1500 ms. Following stimulus presentation, the choice was highlighted for 250 ms after which feedback was presented for 1000 ms.

### Reference-back

Our third task was a replication of the reference-back task as described in Rac-Lubashevsky and Kessler (2016a). Participants compared the stimulus presented on screen to a stimulus held in working memory (the reference stimulus). Two types of trials exist. In comparison trials, the participant only compared the stimulus to the previous reference trial. In reference trials, the participant not only compared the stimulus to the reference stimulus, but subsequently also updated the current stimulus as the new reference stimulus in working memory. Stimuli could be either a white “X” or “O.” A blue frame around the stimulus indicated that the current trial was a comparison trial. A red frame around the stimulus indicated that the current trial was a reference trial. Reference and comparison trials were randomly interleaved. An example trial sequence is shown in Fig. 2.

At the start of each trial, a blank screen was presented for 1000 ms, after which a fixation cross was presented for 1000 ms. Then, the stimulus was presented until a response was made. Participants completed 192 trials per session. One session of the RB task was always paired with a session of the RL-Rev task. The order of the tasks within session and between the two sessions was counterbalanced between participants.

### MSIT

The multi-source interference task is a cognitive control task in which participants have to identify the unique number out of three numbers presented on screen (Bush et al., 2003; Isherwood et al., 2022).

In the “Flanker” condition, the two non-target stimuli have the identity of another valid response. Furthermore, in the “Simon” condition, the position of the stimulus and the identity of stimulus are incongruent (Simon).

In total, we can derive five different conditions from our MSIT design (Fig. 2). In condition 1, the identity of the target is congruent with its position, and the other two stimuli are equal to 0. Since 0 is not a possible response option, there is no response conflict in these trials. In condition 2, the identity of the target and its position differs, resulting in Simon interference. In condition 3, there are Flankers that suggest a different response than the target stimulus, but the position and identity of the target are congruent. In condition 4, there is both Flanker and Simon interference; however, the Flankers point towards a different response than the Simon. Lastly, in condition 5, there is again both Flanker and Simon interference; however, both the Flanker and the Simon interference point towards the same incorrect response.

Participants completed 265 trials per session. On each trial, after the response window, feedback of either “in time” (responses less than 600 ms), “too slow” (responses between 600 and 900 ms), or “very slow” (for responses more than 900 ms) was shown. One session of the MSIT was always paired with a session of the stop-signal task. The order of the tasks within session and between the two sessions was counterbalanced between participants.

**Table 4:** BPIC results for the different models compared for the reference-back experimental data

Model	Type	Response	$Stim_{trans}$	$Typ_{trans}$	BPIC
1	$V_0$	$\delta$	*B*	$V_0$	3642.5
2	$V_0$	$\delta$	$V_0$	*B*	4009.0
3	$V_0$	$\delta$	*B*	*B*	3530.6
4	$\delta$	$\delta$	*B*	$V_0$	3247.8
5	$\delta$	$\delta$	$V_0$	*B*	3866.9
6	$V_0$	$\delta$	*B*	$\delta$	3563.1

Each column in the table corresponds to a manipulation in the data for which there were two conditions. The parameter mentioned in that column entails that for the model corresponding to that row, that parameter was varied between those two conditions. Type refers to reference vs comparison trial. Response refers to the same versus different trials. $Stim_{trans}$ refers to whether the current trial was a switch or repetition of stimulus (“X” or “O”) compared to the previous trial. $Typ_{trans}$ refers to whether the current trial was a switch or repetition of trial type (“reference” or “comparison”) compared to the previous trial

**Table 5:** BPIC results for the different models compared for the multi-source interference task experimental data

Model	Simon	Flanker	Target pos	BPIC
1	*v*	*v*	*v*	$-$21242.8
2	*B*	*v*	*v*	$-$20948.1
3	*v*	*v*	*start*	$-$21326.6
4	*B*	*v*	*start*	$-$21041.1

For the Simon and Flanker conditions (columns), the parameter estimated corresponded to the difference between baseline and that condition. For the Target pos(ition), a different parameter was included for each of the three positions where the target could be in. Position 3 was fixed to 0, for scaling constraints. Note that raising the start point by a fixed amount is equivalent to lower the threshold by a fixed amount within EAMs

## Appendix 2. Model comparisons

Here, we describe the model comparison study we performed for the reference-back (RB) task and multi-source interference task (MSIT). For both tasks, we pooled the data of the two sessions together and estimated different models to that data. Model comparisons were made using the Bayesian predictive information criterion (BPIC; Ando , 2007), rather than Bayes factor estimates using $IS^2$, since in contrast to the joint models, the individual likelihoods were of main interest rather than the group-level distributions, in which case the BPIC is far more computationally efficient. Note that contrary to Bayes factors and marginal log-likelihood estimates, for the BPIC lower is better.

### Reference-back

For the different RB models, we limited our model search space by only comparing models where all manipulations and sequence effects as described in the “Cognitive Modelling” section were captured. Furthermore, we limited ourselves to ten parameter models, which was the minimal amount of parameters to describe all facets of the data, while considering the cognitive plausibility and assumptions of the effect-to-parameter mapping within the framework of evidence accumulation modelling. The BPIC scores are summarized in Table 4.

### Multi-source interference task

For the different MSIT models, the behavioral effects our model was to capture were the Flanker manipulation, the Simon manipulation, and the observed differences between the left and right presentation of the target stimulus. Again, we limited our model search by excluding mappings of these effects to EAM parameters that violated assumptions about these parameters. The BPIC scores are summarized in Table 5.

## Appendix 3. Model fits

Here, we present posterior predictives for the different EAMs we constructed. We summarize accuracy and correct and error response time (RT) distributions of the four different decision-making tasks. For each task, we plot these quantities for the conditions of interest in the experiment. Throughout, we plot the 10th, 50th, and 90th percentile of the RT distributions. The 50th percentile corresponds to the central tendency, and the smaller difference between the 10th and the 50th percentile compared to the difference between the 90th and the 50th percentile summarizes the positive skew generally found in RT distributions. For the RL tasks, we visualize the effect of learning by dividing the trials in bins and plotting the bins along the *x*-axis. All data are collapsed across participants. To assess the overlap between the predictions of the joint model and the models fit individually, we plot the posterior predictives of the joint model in green and of the single, or individually fit, model in blue. We find that the joint model generally predicts very similar behavior as the models fit individually.

For the reinforcement learning reversal task, we found that similar to Miletić et al. (2021), the model underestimates the speed with which participants learn or adjust the stimulus value representations both in terms of response times and accuracy (Fig. 5). Still, the model captures the tendencies in the data well.

The posterior predictives of the reinforcement learning speed-accuracy trade-off task showed that the model underestimates the speed with which participants learn the stimulus value representations, which is specifically noticeable in the accuracy condition (Fig. 6).

In the posterior predictives of the multi-source interference task, we found that the correct RTs across all conditions and the accuracy across the position of the stimulus and the identity of the Flanker match the behavioral data (Fig. 7). We also found that the model underestimated the accuracy when the target stimulus was position 1 and 3. Furthermore, the error response time distributions were consistently overestimated in the model. This misfit is most likely because of the low percentage of errors in the behavioral data.

In the posterior predictives of the reference-back task, we observed slight underestimations in the accuracy across the experimental conditions (Fig. 8). The correct RT distributions generally matched the behavioral data. However, the error RT distributions were mostly underestimated. Again, this is likely because of the low percentage of errors in the behavioral data.
